# Supplementary figures and images for: Reference gene expression stability within the rat brain under mild intermittent ketosis induced by supplementation with medium-chain triglycerides
Source: PLoS One. 2023 Feb 9;18(2):e0273224. doi: 10.1371/journal.pone.0273224 (PMC9910642; doi:10.1371/journal.pone.0273224)

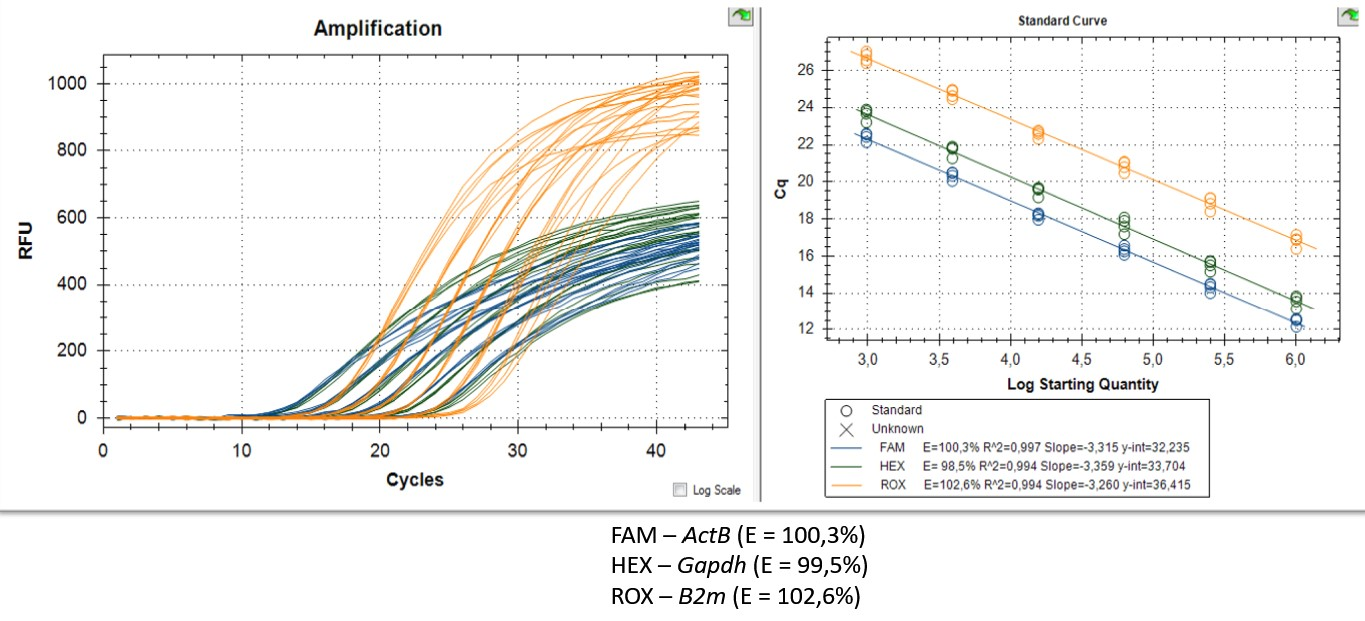

Supplement: S4 Fig — Efficiency was assessed by serial dilution method. Each standard curve was generated from a series of four-fold dilutions of pooled rat brain cDNA samples, collected in the main experiment. (TIFF) [file pone.0273224.s005.tiff]

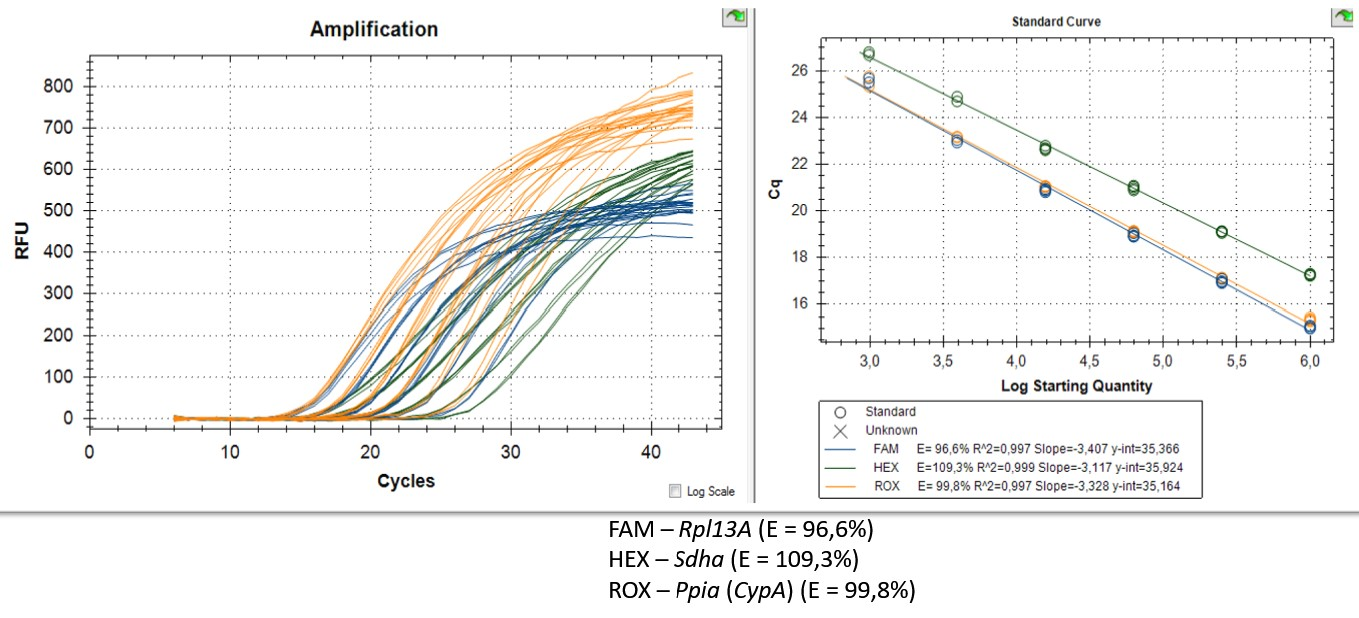

Supplement: S5 Fig — Efficiency was assessed by serial dilution method. Each standard curve was generated from a series of four-fold dilutions of pooled rat brain cDNA samples, collected in the main experiment. (TIFF) [file pone.0273224.s006.tiff]

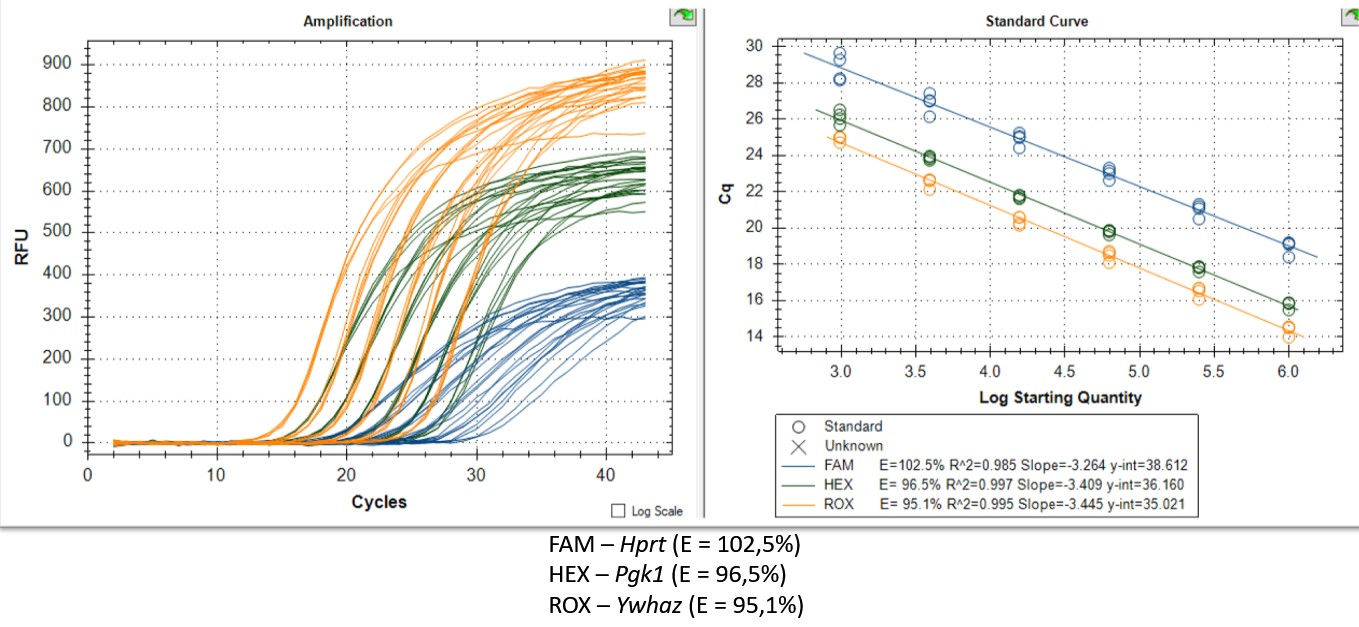

Supplement: S6 Fig — Efficiency was assessed by serial dilution method. Each standard curve was generated from a series of four-fold dilutions of pooled rat brain cDNA samples, collected in the main experiment. (TIFF) [file pone.0273224.s007.tiff]
