# Supplementary material for: Reference gene expression stability within the rat brain under mild intermittent ketosis induced by supplementation with medium-chain triglycerides
Source: PLoS One. 2023 Feb 9;18(2):e0273224. doi: 10.1371/journal.pone.0273224 (PMC9910642; doi:10.1371/journal.pone.0273224)
Supplement: Attachment — Submitted filename: Schwarz_et_al_Response_to_Reviewers.docx [file pone.0273224.s008.docx]

Prof. Dragan Hrncic, MD, PhD
Academic Editor
PLOS ONE

Dear Dr. Hrncic,

Thank you and the reviewers for working on our manuscript and all the valuable comments and suggestions. We have prepared a revised version of the article with the changes marked. Briefly, we have corrected some typos throughout the manuscript and changed the title according to the reviewer's suggestion.
Please find the detailed answers to the reviewers’ comments below.

Reviewer 1
«Title
- Title should be changed to better reflect a full content of the manuscript.
- Maybe, one form of the title could be:” Reference gene expression stability within the rat brain under mild intermittent ketosis induced by supplementation with medium-chain triglycerides”».

We changed the Manuscript title according to the reviewer’s suggestion.

«Abstract
- Please, introduce all abbreviations in appropriate places.
- Avoid starting this section with an abbreviation on the first place of the sentence.
- Address the impact of model of mild ketosis induced by medium-chain triglyceride supplementation and its relationship to genes expression.»

We edited the abstract to mention the full name of the method and added abbreviations’ disambiguation whereby needed (e.g. MCT, RT-qPCR). We also added two sentences describing our model of mild ketosis: this approach allows to reproduce certain neuroprotective effects of the classical ketogenic diet while avoiding its adverse effects. Ketogenic treatment targets multiple metabolic pathways, which may affect the reference gene expression.

«Introduction
- Add a paragraph and explain the importance of selected genes, and describe some other studies which investigated the similar problematic.
- At the end of this section should be placed the aim of this study.»

We rearranged this section, moving the paragraph about the aim of the study to the end of the section. We also added information about the functional properties of the analyzed gene products.

«Material and methods
- In Animals and study design subsection, please add the number of ethical permission.
- Also, your manuscript will benefit by adding of one new figure with experimental timeline, which should be placed in Animals and study design subsection.»
We added the permission number and created a figure with the experimental timeline (Figure 1 in the revised version).

«Results
- In Figures 3-5, for more clarity, colors could be additionally added, but it is not mandatory»

We made colored versions of the graphs (figures 4-6 in the revised manuscript).

Reviewer 2.
1. «Authors need to explain the PCR efficiency of the primers in the manuscript».
We added the information about the range of measured PCR efficiencies to the Method section. The full information is presented in the Supplementary figures S4-S6.

2. «Why were the authors using 2 mL/kg daily? Please give some supportive references.».
We used this dosage to mimic typical low doses used in human studies. We added the supporting references in the Materials and Methods section.
3. «*Line no. : 154: "RefFinder® online tool (https://www.heartcure.com.au/reffinder/)". Delete the website address as it was mentioned once in line 129.»
We deleted the repeated mention of the URL.

4. In general, the authors should be writing medium-chain triglycerides (MCT) as either ‘MCT’ or ‘MCT oil’ in whole article.

We chose MCT for the whole article in the revised version.

5. Though gene expression was normalized with the three reference genes selected, this data still needs to be validated for gene expression with the bottom genes. This further proves the validity of the reference genes selected (Sahu., et al 2018)

We would like to thank Reviewer 2 for this suggestion, but we decided not to change our article in this way. Of course, the calculations for the genes of interest to the unstable genes could be used as a vivid illustration of the biases resulting from incorrect normalization. However, it would be a good approach if we were working with «marker» genes that moderately changed under the analyzed experimental conditions. In our case, we work with a new model, which is not well described in rats. So, we feel that the calculation of custom gene of interest expression normalized to different references would make our article more complicated and would not clarify the results.

We are looking forward for your review of the revised manuscript.

Alexander Schwarz
Ph.D., Research Fellow
Laboratory of  Molecular Mechanisms  of Neuronal Interactions
I.M. Sechenov Institute of Evolutionary Physiology and Biochemistry
St. Petersburg, Russia

Aleksandr.Pavlovich.Schwarz@gmail.com
